# Supplementary material for: Proactive review for people with diabetes in hospital: a cluster randomised feasibility trial with process evaluation, protocol V3.1
Source: Pilot Feasibility Stud. 2024 Jun 11;10:88. doi: 10.1186/s40814-024-01507-2 (PMC11165828; doi:10.1186/s40814-024-01507-2)
Supplement: Supplementary file 2 — Supplementary Material 2. [file 40814_2024_1507_MOESM2_ESM.docx]

**Authors**: Andrea. K. Lake^1,2^, Vishakha Bansiya¹, Katy Davenport¹, Jamie Murdoch^3^, Helen R Murphy^2,1^, Toby Smith^2^, Allan Clark^4^, Antony Arthur^2^

^1^ Clinic 32, Wolfson Diabetes & Endocrine clinic – Box 281, Cambridge University Hospitals NHS Foundation Trust, Hills Road, Cambridge, CB20QQ

^2^ School of Health Sciences, Faculty of Medicine and Health Sciences, University of East Anglia, Norwich, NR4 7TJ

^3^School of Population Health & Environmental Sciences, Faculty of Life Sciences & Medicine, King’s College London, 5th floor, Addison House, Guy’s Campus, London, SE1 1UL

^4^ Norwich Medical School, University of East Anglia, Norwich, NR4 7TJ

Corresponding author and email address:

Andrea Lake^1,2^, NIHR clinical doctoral research fellow

andrea.lake@nhs.net

Tables: 2 Figures: 2

IRAS ref: 302069

Sponsor reference: A096106

Sponsor: Cambridge University Hospitals Foundation Trust

Sponsor address:

Research & Development Department - Box 277

Addenbrookes Hospital

Hills Road, Cambridge, CB2 0QQ

**P**roactive **re**view for **p**eople with **D**iabetes in hospital: a cluster randomised feasibility trial with process evaluation, protocol V3.1

**Short Title**: PREP-D

**Abstract**

**Background**: Diabetes inpatient specialist services vary across the country, with limited evidence to guide service delivery. Currently referrals to diabetes inpatient specialists are usually ‘reactive’ after diabetes related events which are associated with increased risk of morbidity/mortality and increased length of hospital stay. We propose that a proactive diabetes review model of care, delivered by diabetes inpatient specialist nurses may contribute to the prevention of such diabetes related events and resulting increased risk of harm.

**Method**: We will conduct a cluster randomised feasibility study with process evaluation. The Proactive Diabetes Review Model (PDRM) is a complex intervention that focuses on the prevention of potentially modifiable diabetes related harms. All eligible patients will receive a comprehensive, structured diabetes review that aims to identify and prevent potentially modifiable diabetes related harms through utilising a standardised review structure. Reviews are undertaken by a diabetes inpatient specialist nurse within one working day of admission. This differs to usual care where patients are often only seen after diabetes related harms have taken place.

Trial duration will be approximately 32-weeks, with intervention delivery throughout. There will be an initial eight-week run-in phase, followed by a 24-week data collection phase. Eight wards will be equally randomised to either PDRM or usual care. Adult patients with a known diagnosis of diabetes admitted to an included ward will be eligible. Data collection will be limited to that typically collected as part of usual care. Data collected will include descriptive data at both the ward and patient level and glucose measures, such as frequency and results of capillary glucose testing, ketonaemia and hypoglycaemic events. Analysis aims to determine fidelity and acceptability of the intervention and the feasibility of a future definitive trial. While this study is primarily about trial feasibility, the findings of the process evaluation may lead to changes to both trial processes and modifications to the intervention. A qualitative process evaluation will be conducted in parallel to the trial. A minimum of 22 patients, nurses, doctors, and managers will be recruited with methods including direct non-participant observation and semi-structured interviews. The feasibility of a future definitive trial will be assessed by evaluating recruitment and randomisation processes, staffing resources, and quality of available data.

**Discussion**: The aim of this cluster randomised feasibility trial with a process evaluation is to explore the feasibility of a definitive trial and identify appropriate outcome measures. If a trial is feasible and the effectiveness of PDRM can be evaluated, this could inform the future development of inpatient diabetes services nationally.

**Trial registration**: UK Clinical Research Network: 51167; ISRCTN, ISRCTN70402110. Registered 21/02/2022, <https://www.isrctn.com/ISRCTN70402110?q=ISRCTN70402110&filters=&sort=&offset=1&totalResults=1&page=1&pageSize=10>

**Key words:**

Diabetes Mellitus, Hospitalised, inpatient, Diabetes Specialist Nurse, Proactive, Prevention, risk reduction, service model

**Background**

In the UK, it is estimated that one in 15 people currently have diabetes and one in six hospital beds is occupied by someone with diabetes (Diabetes UK, 2019). Hypoglycaemia (blood glucose <4.0 mmol/L) and hyperglycaemia (blood glucose >11 mmol/L) among hospitalised patients with diabetes increases the risk of adverse health-related outcomes such as hospital acquired infections, increased length of stay and increased in-hospital mortality (Marin-Penalver et al, 2016). The National Diabetes Inpatient Audit Group (NaDIA) reported that in England, target glycaemic control was achieved in less than half of all hospital inpatient days. “Good” glycaemic control has been defined by NaDIA as no episodes of hypoglycaemia <4.0 mmol/L and no more than two episodes of hyperglycaemia > 11.0 mmol/L. Additionally, 20% of people with diabetes in hospital experience hypoglycaemia, with 8% of these classed as severe (National Diabetes Inpatient Audit, 2018). Inpatient hypoglycaemia is associated with a three-day increased length of hospital stay and increased risk of in-hospital mortality (Lake, et al, 2019a).

The National Quality board (2018) highlighted the importance of ‘getting the right professional, with the right skills, to review the right patient, at the right time and in the right place’. However, little is known about what interventions are likely to be effective in achieving this. Of the service developments or service evaluations reported, most are complex interventions of which few have undergone methodologically rigorous evaluation (Patel, M, 2018 and Lake et al, 2019b). Many involved additional staff resources (Rajendran, 2015 and Mahto et al, 2008), which might explain the improvement in outcomes reported. None have undertaken a process evaluation or provided an assessment of which of the multiple components of the intervention were likely to be the most effective (Kyi et al, 2019). Improvements in outcomes reported a reduction in mild and severe hypoglycaemia and medication errors, increased staff knowledge, confidence and satisfaction and reduced length of stay (Rajendran, 2015 and Lake et al, 2019b), reduction in delayed discharges, avoidable admissions and inappropriate discharge plans (Mahto et al, 2008) and a reduction in hyperglycaemia and hospital acquired infections (Kyi et al, 2019).

*We propose that a proactive model of care by the diabetes inpatient specialist nurses (DISNs) within one working day of admission has the potential to improve inpatient diabetes management by:*

1. Reducing preventable diabetes related adverse events

2. Improving levels of support offered to non-diabetes specialist colleagues (doctors/ nurses/ AHPs)

3. Working with patients to create their inpatient diabetes management plan

4. Increasing awareness of diabetes management in hospital through increased visibility of the diabetes team on the wards

5. Supporting primary teams to be more knowledgeable and effective in managing diabetes

*The intervention – Proactive Diabetes Review Model*

The Proactive Diabetes Review Model (PDRM) is a complex intervention that combines all the components of the usual care provided by a DISN at the first review and applies the principles recommended by the national quality board by making changes to the timing, focus and ambition of the review. PDRM will be delivered by DISNs for patients with diabetes admitted to hospital. The PDRM consists of a patient centred diabetes assessment undertaken within one working day of admission to an included intervention ward and aims to identify modifiable risk and prevent diabetes related harm (see Table 1). The intervention will consist of a single visit for individual patients, all care after this will fall in line with usual care. Recommendations will be based on best clinical practice, at the discretion of the professional undertaking the review.

Overall responsibility for the patient will remain with the primary ward team (patients admitting specialty doctor and ward nurses, as is currently the case). Therefore, as part of this process, support for the patient’s primary team will be provided through expert recommendations, ‘on the spot’ education as needed, and an increased visibility of the diabetes inpatient specialist nurses on the wards. After the first review the patient will then be triaged into one of two groups: no further review required (re-refer as needed) or ongoing review. PDRM differs from usual care by proactively reviewing all patients with diabetes, not just those who have been referred. Other differences include the structure, timing and aims of the review itself.

| Table 1 - PDRM Intervention components | | |
| --- | --- | --- |
| Category | Detail | Outcome data source |
| An initial bedside assessment of the patient | - Current and historical medications - Feet and injection sites as appropriate - Usual diabetes care provider - Diabetes education history Patient reported history of home glucose level | Reviewing the medical notes and trial paper case report forms |
| A review of routinely collected investigations | - Glucose monitoring - Ketone monitoring where applicable. - HbA1c - Annual review processes, TSH, Urea, creatinine, ACR | Reviewing the medical notes and electronic data exports |
| Recommendations resulting from the shared decision-making process | - Links to relevant policies and guidance where appropriate - Documented in the patients’ medical records | Reviewing the medical notes and trial paper case report forms |
| Provision of any appropriate patient information | - Leaflets - App recommendations - Other patient literature - Signposting to any relevant patient specific resources | Reviewing the medical notes and trial paper case report forms |
| Handover of recommendations to the patient and patient’s primary team | - Admitting speciality doctor - Ward nurses | Reviewing the medical notes and trial paper case report forms |
| Triage | - No further follow-up - Further follow-up by the diabetes specialist team - ongoing reviews will be managed in line with usual care | Reviewing the medical notes and trial paper case report forms |

It is anticipated reviews will take between 15 to 45 minutes depending on complexity. An example of a non-complex review could be an inpatient with diet-controlled diabetes and no expected glycaemic disruption. A complex review for example would include inpatients treated with agents known to cause hypoglycaemia, and/or admitted with complex health needs leading to treatment, such as corticosteroids, known to interfere with glucose levels. Diabetes treatment alone will not be used to indicate individual risk.

*Service evaluation of the model*

In 2018, we undertook a service evaluation of PDRM at the Cambridge University Hospitals NHS Foundation Trust (CUHFT). The service evaluation aimed to refine the model and investigate the potential impact on both the service and patients. All patients with diabetes on eight wards received a proactive review by a DISN within one working day of admission. No adjustments to the model where made. The outcome data that was collected from the intervention wards were compared to the same eight wards, for the same four months (November to February) during the previous year using routinely available retrospective data. Results showed a 30% increase in the number of first reviews being undertaken, with a decrease in hypoglycaemia episodes, shorter length of hospital stay and improved staff satisfaction (Lake et al, 2019b). Kyi et al (2019) also reports potential for improved outcomes utilising a proactive approach but, in both cases, there is no information regarding implementation, contamination or intervention acceptability, which is why further process evaluation is warranted. The definition of acceptability in this trial includes how acceptable the intervention (the timing and content of the diabetes review and form of delivery) and trial methods (processes of recruitment and data collection) are for people with diabetes in hospital, the ward-based staff caring from them and the diabetes inpatient teams delivering the diabetes related care.

Following the MRC framework for developing and evaluating complex interventions, we are now undertaking feasibility testing. The definition of feasibility testing in this trial is to explore the proposed trial design, methods and analysis prior to a definitive trial. Feasibility testing is essential because of what remains unknown (Craig et al, 2013).

***Study Design***

A cRCT comparing the Proactive Diabetes Review Model (PDRM) to usual care (reactive review) and parallel process evaluation (Figure 1). The schedule of enrolment, interventions and assessments are detailed in Figure 2. The SPIRIT checklist (Chan et al, 2013 ) which details the recommended content for a protocol has been included as Additional Paper 1.

A cRCT randomly allocates an intervention to a cluster or group of individuals within a cluster (Weijer et al, 2011). Cluster randomised controlled trials are appropriate when it is not possible to randomise at an individual level. In this study, individual randomisation was not appropriate due to the risk of contamination between and across individuals either directly with each other or through the staff providing their care (Baxter et al, 2020; Dron et el, 2021)

***Feasibility Trial***

*The Trial*

Eight wards will be recruited with four randomised to receive the PDRM intervention and four usual care. As this is a feasibility trial, a formal power calculation is not indicated and has not been undertaken. The sample size of eight wards is a pragmatic choice to allow sufficient recruitment across inpatient specialities, whilst not recruiting wards unnecessarily. All wards will undergo an eight-week run-in period to allow a period of ‘settling in’. This aims to allow all the PDRM DISNs time to become familiar with the process and trial related activities prior to data collection. A 24-week intervention/data collection period will then begin.

Figure 2**.** Schedule of enrolment, interventions, and assessments.

|  | **STUDY PERIOD** | | | | | |
| --- | --- | --- | --- | --- | --- | --- |
|  | **Enrolment** | **Allocation** | **Post-allocation participant screening and recruitment (run-in phase)** | **Post-allocation participant screening and recruitment (data collection phase)** | **Participants follow up** | **Close-out** |
| **TIMEPOINT (weeks)** | ***-10*** | **0** | ***0-8*** | ***9-32*** | ***9-34*** | ***34*** |
| **ENROLMENT:** |  | | | | | |
| **Divisional Eligibility** | X |  |  |  |  |  |
| **Ward Eligibility** | X |  |  |  |  |  |
| **Ward allocation** |  | X |  |  |  |  |
| **Participant eligibility screening and consent** |  |  | X | X |  |  |
| **INTERVENTIONS:** |  | | | | | |
| ***PDRM*** |  |  |  | |  |  |
| ***Care as usual*** |  |  |  | |  |  |
| ***Process evaluation*** |  |  |  | | |  |
| **ASSESSMENTS:** |  | | | | | |
| ***Ward baseline data*** |  | X |  |  |  |  |
| ***Trial participant run-in data collection*** |  |  | X |  |  |  |
| ***Feasibility, process, and ward level outcome data collection*** |  |  |  | X | X |  |
| ***Trial participant outcome data (collected post discharge)*** |  |  |  | X | X |  |
| ***End of data collection*** |  |  |  |  |  | X |

*Trial population and Eligibility*

There will be eligibility and recruitment at two levels: ward (clusters) and patients (within clusters).

Wards

Eight general adult medical and surgical wards will be recruited. Specialist diabetes wards, day-case units, maternity, and paediatric wards will be excluded.

Recruitment of wards will happen at divisional and ward level. A division is made up of closely allied specialities and services that operate within a common governance structure to deliver seamless patient pathways. A specific information sheet is developed for divisional leads and ward managers. Divisional lead approval will be sought. Following approval, ward managers and clinical leads of wards identified as eligible for inclusion, will be approached for recruitment.

Patients

Adult inpatients with a diagnosis of diabetes documented on their problem list and admitted to one of the included wards will be eligible. They will be identified via a daily report run by the diabetes specialist team administrator. Inpatients already under the care of the specialist diabetes team in hospital will be excluded. Eligible patients will be approached during their hospital stay for recruitment and to obtain informed consent for use of their data and provided with an information sheet.

*Randomisation*

Randomisation will be stratified by ward type (medical or surgical). Wards will be randomly allocated to receive either usual care or the PDRM intervention. The Ralloc procedure in Stata® will be used. Block randomisation will limit imbalance in trial arms. Randomisation will take place following ward recruitment and study team training.

*Usual care*

Wards randomised to receive usual care will receive a reactive review by a DISN on receipt of a referral by a member of the primary team (patients admitting speciality doctor and ward nurses) or if highlighted through the daily high-risk report. Both are current usual practice in the participating site. The structure of the review undertaken, recommendations made and frequency of follow up will be as per current local practice.

Patient identification

A daily electronic report will identify all admitted patients with diabetes on an included ward. Once identified, for patients on an intervention ward, the DISN will complete an initial remote assessment to establish whether a PDRM review is appropriate. Examples of potential exclusions would be if a diabetes medical review is required (where there is diagnostic uncertainty or complex medical needs), or if a PDRM is not required (where a patient is in the last hours of life or due for imminent discharge and no diabetes related concerns).

*Approach and consent*

There are known practical challenges to receiving informed consent at an individual patient level in a cluster randomised design (Campbell et al, 2012 and McRae et al, 2011). This has been explored through a review of other cluster randomised designs (Weijer et al, 2011, Weijer et al, 2015, Lignou, 2017 and McRae et al, 2011) and seeking patient, public and expert opinion through patient and public involvement activities. The consent process as described has been designed to meet both the legal and ethical requirements for research while also considering potential risks associated with the methodology used.

Individual patient informed consent will be sought for the collection of patient identifiable data. Permission for the delivery of the PDRM intervention in the clinical area will be obtained from the ward manager and clinical lead prior to ward level randomisation.

Receipt of the PDRM intervention does not equate to participant inclusion in the study and declining the PDRM intervention does not exclude patients from being eligible for the trial.

By separating these processes, patients will be able to receive (or refuse) a diabetes review in line with usual clinical care, even if they do not consent to their data being included in the study. Equally, patients can opt to have their data included even if they did not wish to receive the PDRM intervention.

*Outcomes*

Given the feasibility design of this study, outcomes have been split into two categories: (1) feasibility and process; and (2) trial.

*Feasibility and process outcomes*

1. Proportion of approached wards that agree to participate

2. Proportion of eligible patients that are recruited

3. Proportion of eligible patients on the ward seen by a diabetes specialist during their admission

4. Time from admission to an included intervention ward to receiving a review by a diabetes specialist

*Trial outcomes*

1 Number of days, per patient admission, where glucose testing completed

2 Incidence of hypoglycaemia (rate of biochemical hypoglycaemic episodes defined as blood glucose <4.0 mmol/L during hospital stay)

3. Incidence of hyperglycaemia >15.0 mmol/L

4. Incidence of positive ketones (blood ketone ≥1.5 mmol/L)

5. Incidence of hospital acquired foot ulceration

6. Length of hospital stay (total number of inpatient days)

The feasibility of a future definitive trial will be determined by:

1. Evidence that recruitment at both the cluster and individual level can be achieved
2. Evidence that the intervention can be delivered. The proportion of patients who received the proactive review
3. Evidence of availability of trial outcome data

We will analyse our feasibility data for recruitment and intervention delivery against the progression criteria as set out in table two. Where the criteria set out in the green column is met, the study will be considered feasible. If only the criterion in the amber column is met, feasibility will be dependent on whether a definitive trial could address this methodologically, drawing on the process evaluation data. If the amber criteria are not met the study will not be considered feasible. For progression to a definitive trial, the findings must demonstrate that all five green or amber criteria have been met, with strategies to address where we have not met green criteria.

| Table 2: Progression criteria | | | |
| --- | --- | --- | --- |
|  | GREEN | AMBER | RED |
| Cluster recruitment to target (8 wards) | 90-100% | 70-89% | <70% |
| Participant recruitment: proportion of approached and eligible patients consenting to participate | 90-100% | 70-89% | <70% |
| Intervention delivery: proportion of patients that received the intervention (intervention arm participants only) | 90% to 100% | 70 to 89% | <70% |
| Median time from identification to receiving a review by a diabetes specialist (intervention arm participants only) | Within one working day | One-three working days | Four or more working days |
| Trial outcome data available | 90 to 100% | 70 to 89% | <70% |

*Data collection*

Descriptive and outcome data will be collected throughout the eight-week run-in period and 24-week intervention and follow up period. To reduce patient burden, a pragmatic approach to data collection will be adopted by restricting all quantitative data to information routinely collected as part of clinical care. Data will be extracted after the participant has consented and the episode of care completed (i.e. after hospital discharge). Data extraction will include a mix of reviewing the medical notes, electronic exports, and trial paper case report forms. Ward levels data will be provided by the ward managers through a spreadsheet requested monthly.

Descriptive statistics and outcome measures will be described at baseline (run in phase) and trial end. Data will be collected for the duration of a participant’s hospital stay. Their inclusion in either the baseline or trial end analysis will depend on the timepoint of their admission during the trial. Baseline analysis will use the data of participant’s admitted during the run-in phase (weeks 0-8) and outcome analysis the data of those participants admitted during weeks 9 to 36.

*Statistical analysis*

Groups at baseline will be described using summary statistics at both ward and patient level. Changes from baseline will be described within and between the two trial arms from run-in to follow-up periods. The purpose of the analysis is (1) to describe potential effect via confidence intervals allowing for the clustered design, (2) as a way of trialling an analytic plan for a future definitive trial if the proposed study suggests one is feasible and (3) the extent and distribution of missing data will be reported for consideration as to how to manage this in a definitive trial. Statistical analysis will be completed using the latest version of STATA® with the trial statistician (AC). No interim analysis of trial outcomes between arms is planned.

*Sample size*

The trial outcome measures are designed to address the research aims and objectives. This exploratory feasibility trial is not statistically powered to detect superiority of treatment effects. A cluster level recruitment target of eight randomised wards on a 1:1 basis has been chosen to allow sufficient recruitment and diversity in the population to answer the study questions.

A participant level recruitment objective has not been set as this is one of the feasibility outcomes. However, based on the service evaluation we anticipate 500-800 potentially eligible participants to be identified with 250-500 being recruited.

***The Process Evaluation:***

A parallel process evaluation, underpinned by ethnographic methodology will investigate delivery and acceptability of the PDRM intervention to identify how to refine the intervention and trial design, to optimise implementation in a future definitive trial (Moore et al, 2015). Qualitative methods will be used, including non-participant observations and de-brief interviews of PDRM interactions, and semi-structured interviews with patients and professionals.

*Process evaluation specific objectives*

1. Assess the extent to which the PDRM is delivered as intended
2. Assess acceptability of the PDRM intervention
3. Assess trial processes including recruitment and consent
4. Identify any potential sources of contamination in the care as usual arm
5. Identify contextual barriers and facilitators of delivery
6. Identify outcomes that are important to patients

*Process evaluation population and eligibility*

Patients

Eligibility for the process evaluation and feasibility trial is identical. Consent to take part in the process evaluation is separate to, but conditional on, participation in the feasibility trial. All patients eligible for participation in the process evaluation will be approached at the bedside by the Chief Investigator (AL). A minimum of eight patients will be purposefully recruited for semi-structured interviews, and four for direct observation, from across the two trial arms, including those requiring a simple review and those requiring a complex review. The aim is to obtain maximum variation across both forms of diabetes care delivery. The recruitment target was decided as a minimum for patients for pragmatic reasons whilst ensuring diversity. Pragmatic reasons include capacity of the Chief Investigator to undertake the process evaluation activities and possible challenges in recruiting eligible participants across all the various recruitment criteria.

Healthcare professionals

Nurses and doctors from included wards will be eligible, along with all members of the DISN team responsible for delivering the PDRM intervention. As key stakeholders, divisional leads with service development, budget assignment and strategic decision-making responsibilities and ward managers will also be eligible and known as managers for the purpose of this protocol.

A minimum of eight doctors and nurses who work on included wards will be recruited. Purposeful recruitment will take account of experience, seniority, and specialism within both trial arms to ensure a variety of perspectives. A minimum of two divisional leads will be recruited from any included division. A minimum of four DISNs will be included for the direct observation of the PDRM intervention. All HCPs (Health care professionals) will be invited to take part either through invitation face to face or via their work email address.

*Process evaluation data collection methods*

Observations of PDRM delivery and debrief interviews

Observations will be undertaken by the Chief Investigator (AL) to understand the extent to which the PDRM is delivered as intended and if any contextual factors are influencing the PDRM review, such as the ward environment, review interruptions or staff along with the action, reaction, and interactions of the DISNs and the patients (Craig et al, 2013). Recruitment to the observations can take place from Week 8 of the trial onwards and will be undertaken on an *ad hoc* basis for pragmatic reasons. The observations require both eligible and willing patients and DISNs. Recruitment aims to include patients with varying complexities of diabetes management.

Both the DISNs and the patients involved in the observed PDRM interaction will be separately invited to take part in a short debrief interview as soon as is feasible following the review. The aim is to further develop understanding and seek points of clarification from the observation and elicit both patient and staff reflections of the PDRM review. Observations will take place at the bedside and will be voice recorded with consent and then transcribed verbatim. An observation guide will be used.

Semi-structured interviews

The semi-structured interviews will aim to explore the patients’, ward doctors’, ward nurses’, and managers’ experience of diabetes support services offered within the hospital in both arms. These aim to gain a deeper understanding of their experiences of diabetes management in the hospital environment, identify any potential sources of contamination between trial arms, obtain views of the diabetes care received in hospital, the study recruitment and consenting process, and what diabetes related outcomes are important for them.

For those nurses, doctors and ward managers working on the intervention wards, this will include the acceptability of the PDRM and elicit their views on how to refine the intervention. For those staff working on the care as usual arm this will focus on their experience of diabetes support services within the hospital.

Interviews with managers will be conducted to understand contextual barriers and facilitators of delivering the intervention, focusing on how inpatient diabetes management services work together, their experiences of developing inpatient services and how the PDRM can be integrated into routine diabetes care.

The chief investigator will conduct all the interviews undertaken for the process evaluation in a private room on the hospital site or via NHS approved online meetings platform. Interviews, either face to face or via online meeting platforms will be voice recorded with consent and transcribed verbatim. A semi-structured interview guide will be used.

*Process evaluation analysis*

Data analysis will focus on identifying key themes in intervention implementation and delivery of trial processes. Transcripts from both the observations, debrief and semi-structured interviews will be inductively analysed using the principles of thematic analysis (Braun and Clarke, 2006), using the data software package NVivo®. A constant comparison approach will be adopted, working iteratively between data obtained from different interviewees to test out analytical themes, including searching for disconfirming cases and returning to transcripts to ensure authenticity (Connelly, 2016 and Creswell, 2013 pp.179-187). Observational field notes will be analysed to provide a description of how PDRM is delivered, challenges encountered, how patients responded, as well as identifying and explaining variation in content and delivery of the intervention components. Observational and interview data will then be triangulated (Castleberry and Nolen, 2018) to explore potential reasons for any variations in implementation of the PDRM and trial processes, reasons for any observed contamination, to refine and optimise the PDRM delivery and methods used in a future definitive trial.

**Data management and safety reporting**

Participants’ data will be protected in line with the current General Data Protection Regulation (Information Commissioners Office, 2018) and the data controller will be the sponsor (Cambridge University Hospitals NHS Foundation Trust). All patient level data will be requested via Trust employed staff and limited to hospital number and basic demographic details before being electronically transferred into an anonymised form. All data related activities will take place via NHS Trust authorised computers.

For the process evaluation all participants will be allocated a numerical identifier. A participant log will be used to link the participant’s name to their numerical ID should their data need to be withdrawn later. All transcriptions and notes will use the numerical identifier to ensure that participants cannot be identified.

Given this is an in-hospital study, it is expected that all participants will be acutely unwell and at increased risk of medical deterioration. As a result, the traditional definition of SAEs (Serious Adverse Events) may lead to over reporting in this low-risk study of a new service model, so a clear definition of SAEs relating to this study has been provided and is in line with HRA safety reporting requirements. All reportable safety forms will be reported in line with HRA requirements and reviewed by the TSC at the planned intervals set out in the TSC terms of reference.

**Trial organisation and approvals**

This study is being undertaken as part of a National Institute of Health Research Clinical Doctoral Fellowship held by AL (NIHR300530). The trial is sponsored by Cambridge University Hospitals NHS Foundation Trusts. Approvals were gained by the Cambridge central research ethics committee and health research authority on 13^th^ January 2022 (REC (Research Ethics Committee) ref: 21/EE/0275). The study was registered on the ISRCTN registry on 21^st^ February 2022 (ISRCTN70402110).

**Patient and public feedback**

Patient and public feedback has informed both the need and development of the PDRM model. The Group for Research and Clinical Experience in Diabetes (GRACED) was consulted in April and June 2018 and March 2019. GRACED is a local patient group that has and continues to be a collaborator on the development of the PDRM. The group is made up primarily of people with type 1 diabetes. The Diabetes UK group is a larger patient group, primarily made up of older people with type 2 diabetes and was consulted in August 2018 and March 2019. Additionally, the chief investigator held a focus group in April 2019 through the established PPI group at Cambridge University Hospitals Foundation Trust.

Personal experiences of receiving incorrect treatment and support for their diabetes was reported. One person reported experiencing hypoglycaemia on the ward and being offered inappropriate treatment. Others reported feeling the “need to support fellow patients on the ward” as the ward staff’s knowledge of diabetes management was lacking. Of those PPI participants that reported having problems or concerns, none had been offered a review by a DISN, despite having sub-optimal inpatient diabetes control. Both people with diabetes and members of the public supported the idea of investigating the PDRM and recognised its potential benefits.

The trial steering committee has two expert-by-experience members who will be actively involved in the trial steering committee management and oversight of the project.

**Discussion**

Due to the complex nature of the hospital system and the heterogeneous population we expect practical and operational challenges. The feasibility design of this trial aims to allow for a better designed definitive trial, if the study provides evidence of its viability. A limitation is that no pre-defined criteria to assess success have been set. This will be explored as part of the analysis to inform the development of success criteria moving forward. It is possible that patients included in the study will have changes in their clinical situation due to their underlying acute illness. It is expected that there may be a risk of contamination between included wards as patients may also transfer from ward to ward during their admission. This will be captured as part of the data collection process and explored as part of the process evaluation.

Additionally, it is only practical to train all the DISNs to provide the PDRM. Therefore, there is a risk of contamination as the training provided and structured guidance on the review procedure will transfer over to practice when a patient is referred from the care as usual arm. This cannot be avoided within this feasibility design. This may influence the structure of the review provided in the care as usual arm by the DISN, but it is not anticipated it will influence the practice of the primary teams (patients admitting specialty doctor and ward nurses) or increase referrals to the inpatient diabetes specialist team. Contamination will be explored as part of the process evaluation.

**Trial Status**

The current protocol in use at the time of writing was version 2.0 dated 12^th^ January 2022. Recruitment began on the 25th of April 2022 and will end on the 2^nd^ December 2022.

**Declarations**

*Ethics approval and consent to participate*

Ethics and Health research authority approval was received on 13th January 2022 from the Cambridge central research ethics committee, REC ref: 21/EE/0275.

*Consent for publication*

Not applicable

*Availability of data and materials*

Any requests for access to the data or resources should be made to the corresponding author. All requests will be considered on an individual basis and will be approved by the trial steering committee, in line with sponsor regulations.

*Competing interests*

The authors declare no competing interests

*Funding*

Andrea Lake, Clinical Doctoral Research Fellow (NIHR unique award identifier: NIHR300530) is funded by Health Education England (HEE) / NIHR for this research project. The views expressed in this publication are those of the author(s) and not necessarily those of the NIHR, University of East Anglia, Cambridge University NHS foundations Trust, NHS or the UK Department of Health and Social Care.

*Authors' contributions*

All authors contributed to the study and protocol development. AL is the study lead and drafted the manuscript. All authors revised the manuscript and agreed the definitive version.

*Acknowledgements*

The authors would like to thank the members of the trial steering committee, our public and the patient involvement groups, especially GRACED for their contributions to the protocol and development of the study. We would also like to thank the NIHR for supporting the lead author through a personal fellowship, the University of East Anglia, Cambridge University Hospitals NHS Foundation Trust for sponsoring the study, all its patients and staff in the participating divisions and wards and the Wolfson Diabetes & Endocrine Clinic, and the ongoing support from the Clinical Research Network (Eastern).

*Authors' information (optional)*

^1^ Cambridge University Hospitals NHS Foundation Trust, ^2^ University of East Anglia and ^3^ Kings College London

**List of Abbreviations and relevant definitions**

| AE | Adverse Event |
| --- | --- |
| CAU | Care as usual |
| CDRF | Clinical Doctoral Research Fellowship |
| CI | Chief Investigator |
| cRCT | Cluster Randomised Controlled Trial |
| CRF | Case Report Forms |
| CRN | Clinical Research Network |
| Coi | Conflict of Interest |
| CONSORT | Consolidated Standards of Reporting Trials |
| DKA | Diabetic Ketoacidosis |
| DM | Diabetes Mellitus |
| DOT | Diabetes Outreach Team |
| DISN | Diabetes Inpatient Specialist Nurse |
| eGFR | Estimated Glomerular Filtration rate |
| EPIC | Electronic hospital patient record |
| GCP | Good Clinical Practice |
| GDPR | General Data Protection Regulation |
| HbA1c | Glycated haemoglobin (A1c) |
| HCP | Health Care Professional |
| HRA | Health Research Authority |
| ITT | Intention to treat |
| NIHR | National Institute for Health Research |
| PDRM | Proactive Diabetes Review Model (Intervention) |
| PPI | Patient and Public Involvement |
| RCT | Randomised Controlled Trial |
| R&D | Research & Development |
| REC | Research Ethics Committee |
| SAE | Serious Adverse Event |
| T1DM | Type 1 Diabetes Mellitus |
| T2DM | Type 2 Diabetes Mellitus |
| TSC | Trial Steering Committee |

**Reference List**

Baxter. R, Murray. J, O’Hara. J. K, Hewitt. C, Richardson. G, Cockayne. S, Sheard. L, Mills. T, Lawton. R and PACT research team (2020) Improving patient experience and safety at transition of care through the your care needs you (YCNY) intervention: a study prototocol for a cluster randomised controlled feasibility trial, *Pilot and Feasibility Studies,* vol 6, 123, https://doi.org/10.1186/s40814-020-00655-5

Braun, V., & Clarke, V. (2006). Using thematic analysis in psychology. *Qualitative Research in Psychology, 3*(2), 77–101.

Campbell. M. K., Piaggio. G., Elbourne. D. R. and Altman. D.G., (2012) Consort 2010 statement: extension to cluster randomised trials, *BMJ*, vol 345, pp. e5661.

Castleberry. A, and Nolen. A, (2018) Thematic analysis of qualitative research data: Is it as easy as it sounds?, *Currents in Pharmacy Teaching and Learning*, vol 10, no 6, pp. 807-815.

Chan. A.W., Tetzlaff. J.M., Altman. D.G., Laupacis. A, Gøtzsche. P.C., Krleža-JerićK,Hróbjartsson. A., Mann. H., Dickersin. K., Berlin. J.A., Doré. C.J. (2013) SPIRIT 2013statement: defining standard protocol items for clinical trials. Ann *InternMed*, vol 158, no 3, pp.200–7.

Connelly. L. M. (2016) Trustworthiness in Qualitative Research, *MEDSURG* Nursing, vol. 25, no. 6, pp. 435–436.

Craig. P., Dieppe. P., Macintyre S., Michie S., Nazareth I. and Petticrew. M. (2013) Developing and evaluating complex interventions: the new Medical Research Council guidance, *International Journal of Nursing Studies*, vol 50, no. 5, pp. 587-592.

Creswell. J. (2013) Qualitative Inquiry & Research Design, 3rd ed. Los Angeles: SAGE.

Diabetes UK. (2019) *“Us, diabetes and a lot of facts and stats,”* [Online], Accessed Jan 2019. Available: https://www.diabetes.org.uk/resources-s3/2019- 02/1362B_Facts%20and%20stats%20Update%20Jan%202019_LOW%20RES_EXTERNAL.pdf

Dron. L, Taljaard. M, Cheung. Y.B., Grais. R, Ford. N, Thorlund. K., Jehan. F, Nakimuli-Mpungu. E, Xavier. D, Bhutta. Z. A, Park. J. H and Mills. E J (2021) The role and challenges of cluster randomised trials for global health, *The Lancet*, vol 9, no 5, ppE701-E710.

Health Education England. (Accessed: 25-Apr-2019). Health Education England [Online]. Available at: <https://www.hee.nhs.uk/>

Health Research Authority, “Approvals and amendments” [Online], 16-Apr-2019. Available: <https://www.hra.nhs.uk/approvals-amendments/>

Imison. C., Sonola. L., Honeyman. M., and Ross. S. (2014.) *“The reconfiguration of clinical services What is the* *evidence?,”* The Kings Fund [Online]. Available at: <https://www.kingsfund.org.uk/publications/reconfiguration-clinical-services>

Information Commissioners Office, “Guide to the General Data Protection Regulation” [Online], 25-May-2018. Available: https://www.gov.uk/government/publications/guide-to-thegeneral-data-protection-regulation

Kyi. M., Colman. P.G., Wraight. P.R., Reid. J., Gorelik. A., Galligan. A., Kumar. S., Rowan. L.M., Marley. K.A., Nankervis. A.J., Russell. D. M., and Fourlanos. S. (2019) Early Intervention for Diabetes in Medical and Surgical Inpatients Decreases Hyperglycemia and Hospital-Acquired Infections: A Cluster Randomized Trial., *Diabetes Care*, vol. 42, no. 5, pp. 832–840.

Lake. A., Byrne. C., and Murphy. H. (2019a) The impact of hypoglycaemia during hospital admission on health‐related outcomes for inpatients with diabetes: A systematic review, *Diabet. Med*., vol. 36, no. S1, pp. 130–131.

Lake. A., Baillie. J., Durgahee. N., Jeffs. A., Lavender. M., Taylor. C., Bansiya. V., and Davenport. D., (2019b) Service evaluation of the impact of adopting a proactive review process by the Diabetes Outreach Team on patients admitted to hospital with a known diagnosis of diabetes, *Diabet. Med*., vol. 36, no. S1, pp. 77–78.

Lignou. S (2017) Informed consent in cluster randomised trials: new and common ethical challenges, *Journal Medical* *ethics,* vol 44, pp 114-120

Mahto. R., Venugopal. H., Vibhuti. V. S., Mukherjee. A., Cherukuri. V., Healey. B., Baskar. V., Buch. H. N., and Singh. B. M. (2008) The effectiveness of a hospital diabetes outreach service in supporting care for acutely admitted patients with diabetes, *QJM*, vol. 102, no. 3, pp. 203–207.

Marin-Penalver. J.J., Martin-Timon. I., and Del Canizo-Gomez. J. (2016) Management of hospitalized type 2 diabetes mellitus patient, *Journal of Translational internal medicine*, vol 4, no 4, pp. 155-161.

McRae. A. D., Weijer. C., Binik. A., Grimshaw. J. M., Boruch. R., Brehaut. J. C., Donner. A., Eccles. M. P., Saginur. R., White. A. and Taljaard. M. (2011) When is informed consent required in cluster randomized trials in health research?, *Trials*, vol 12, no 202, pp. 1-13.

Moore, G. F., Audrey, S., Barker, M., Bond, L., Bonell, C., Hardeman, W., Moore, L., O'Cathain, A., Tinati, T., Wight, D., & Baird, J. (2015). Process evaluation of complex interventions: Medical Research Council guidance. *BMJ (Clinical research ed.)*, *350*, h1258. https://doi.org/10.1136/bmj.h1258

National Diabetes inpatient Audit (2018) *“National Diabetes Inpatient Audit: are hospitals providing good quality care?”,* [Online], Jun 2018. Available at: <https://www.diabetes.org.uk/resourcess3/2018-06/NaDIA_%20Summary_v6.pdf?_ga=2.204443347.250004555.1529573739-1251873676.1529573739>

National Quality Board. (2018) *“Supporting NHS providers to deliver the right staff, with the right skills, in the right place at the right time: safe sustainable and productive staffing”* 2904770 NQB Guidance v1_2_with links A [Online], 12-Oct-2018. Available at: <https://www.england.nhs.uk/wp-content/uploads/2013/04/nqb-guidance.pdf>

Patel. M. (2018) ‘Marathon not a sprint’: 10 years of inpatient diabetes care in Southampton, England, *Pract. Diabetes*, vol. 35, no. 6, p. 199–202a.

Rajendran. R., Kerry. C., Round. R. M., Barker. S. Scott. A., Rayman. G. and the DICE team. (2015) Impact of the Diabetes Inpatient Care and Education (DICE) project and the DICE care pathway on patient outcomes and trainee doctor knowledge and confidence, *Diabetic Medicine*, vol 32, no 7, pp. 920-924.

Vaismoradi. M., Turunen. H. and Bondas. T. (2013) Content analysis and thematic analysis: Implications for conducting a qualitative descriptive study, *Nursing& Health Science*, vol 15, pp. 398- 405.

Weijer. C, Grimshaw. J .M, Taljaard. M, Binik. A, Boruch. R, Brehaut. J .C, Donner. A, Eccles. M. P, Gallo. A, McRae. A. D, Saginur. R and Zwarenstein. M (2011) Ethical issues posed by cluster randoimised trials in health reseatch, *Trials,* vol 12 doi: 10.1186/1745-6215-12-100

Weijer. C, Taljaard. M, Grimshaw. J. M, Edwards. S. J. L, Eccles. M. P, and The Ottowa Ethics of Cluster Randomised Trial Consensus Group, *Research Ethics,* vol 11, no 1, pp52-60
